# Increasing Competitiveness through the Implementation of Lean Management in Healthcare

**DOI:** 10.3390/ijerph17144981

**Published:** 2020-07-10

**Authors:** J. Carlos Prado-Prado, Jesús García-Arca, Arturo J. Fernández-González, Mar Mosteiro-Añón

**Affiliations:** 1 Industrial Engineering School, University of Vigo (Spain), 36310 Vigo, Spain; jcprado@uvigo.es (J.C.P.-P.); ajfdez@uvigo.es (A.J.F.-G.); 2 Sleep Unit Department, Complexo Hospitalario Universitario de Vigo (Spain), 36213 Vigo, Spain; Mar.Mosteiro.Anon@sergas.es

**Keywords:** lean management, lean healthcare, employee participation

## Abstract

The main aim of this paper was two-fold: first, to design a participative methodology that facilitates lean management implementation in healthcare by adopting the action research approach; second, to illustrate the usefulness of this methodology by applying it to the sleep unit of a public hospital in Spain. This methodology proposes the implementation of lean management in its broadest sense: adopting both lean principles and some of its practical tools or practices in order to achieve competitive advantage. The complete service value chain was considered when introducing changes through lean management implementation. This implementation involved training and involving staff in the project (personnel pillar), detecting and analysing “waste” in value chain processes (processes pillar) and establishing control and measurement mechanisms in line with objectives (key performance indicators pillar) and putting in place improvement actions to achieve these objectives. The application of this methodology brought about an improvement in the management of patient flow in terms of effectiveness, efficiency and quality but also an internal transformation towards lean culture.

## 1. Introduction

Over the past few years in the healthcare sector, changes have occurred that have had a considerable impact on society. Steps have been taken towards an increasing focus on the patient. Management models have been steadily implemented which have given an increasingly direct connectivity with the patient; some examples include telemedicine, electronic clinical history and the Health 4.0 phenomena [1]. Moreover, regulatory measures are being put in place to deal with a contracting economy and an ageing population, where the number of chronic patients is continually increasing. The aim of these measures is to maintain the quality of service without incurring non-assumable costs.

Hospitals and other health services providers are under threat from this changing environment with increasing demands from patients and decreasing budgets. At the same time, they face the challenge of meeting the needs not only of the various stakeholders involved (including governments, healthcare professional bodies, healthcare product and service suppliers and insurance companies) but also society as a whole, particularly during crises such as the COVID-19 outbreak, which test the resilience of health systems all over the world.

Service providers need to respond to challenges by examining how to increase their possibilities of survival by achieving a competitive advantage, especially when they belong to public health services [2]. As is often the case, it is in times of crisis when the need arises to dedicate resources and effort to innovate in an organisation’s management. Accordingly, more and more hospitals have redesigned their internal management with respect to processes, resources and objectives, gearing themselves towards more effective and efficient management, and indeed enhancing the quality of service. Academic literature shows cases where hospitals have achieved this thanks to the adoption of management approaches coming from industrial sectors which with minor nuances or differences among them seek improved efficiency and efficacy of processes and productive systems [3,4,5,6,7,8,9,10,11,12,13]; these approaches include continuous improvement, kaizen, total quality management (TQM), just in time (JIT), six sigma and, particularly, lean management. One key to success in the adoption of these approaches is for affected staff to participate directly [14,15,16,17].

In the healthcare sector, these approaches are seen as innovative, bringing about a radical change in the way things have been done to date. In fact, Walley [18] points out that when the service sector is compared to the industrial sector, it is widely felt that the service sector, and healthcare in particular, is lagging in terms of adopting new management innovations and improvements. For many years, professional medical knowledge was considered sufficient to ensure quality and safety in the delivery of healthcare services.

However, today’s healthcare delivery systems are complex, calling for further organisational awareness in order to provide the appropriate medical care along the entire patient pathway, without incurring extra costs and generating savings. Consequently, the problems with healthcare today are not only clinical but largely organisational. Given the current complexity in the nature of healthcare and its environment, all people involved should participate in the analysis, diagnosis and redesign of the processes for offering a service with a view to managing the available resources simply, effectively and efficiently, all the while in keeping with patient needs and stakeholder expectations.

In this context, this paper’s research question is to analyse whether it is possible to define and implement a participative methodology that systematically seeks to redesign processes in health services, by applying the scientific action research approach from a lean management perspective. The action research approach could be defined as:

*“an emergent inquiry process in which applied behavioural science knowledge is integrated with existing organizational knowledge and applied to address real organizational issues. It is simultaneously concerned with bringing about change in organizations, in developing self-help competencies in organizational members and adding to scientific knowledge. Finally, it is an evolving process that is undertaken in a spirit of collaboration and co-inquiry”*.

[19]

The first scientific contribution of this paper is to propose a new participative methodology for deploying lean principles in health services. This methodology allows an overall, integrated vision of the value chain of the service on offer to the patient (patient flow), involving all corresponding activities, personnel, technical resources, information and objectives. The second major contribution is to apply that methodology by following the action research approach, which is little used in the scientific literature when dealing with lean management implementation, particularly, in health services.

This paper has six sections. After this brief introduction, the second section explains the authors’ methodological proposal. The third section describes the practical application of the methodology in a Spanish hospital unit. The fourth section presents the main results obtained and the fifth section develops the discussion of the results with due regard to the limitations of the study and some future lines of research that would give continuity to the study. Finally, the conclusions are presented in the sixth section.

## 2. Materials and Methods 

As mentioned in the introduction, the main research question here analyses whether it is possible to design and implement a participative methodology from a lean management perspective that systematically seeks to redesign processes in health services by applying the scientific action research approach. According to Westbrook [20], the action research approach began when some authors started showing an interest in the field of social psychology in the 1940s. They used it to develop research that was not only useful for firms and organisations but that also promoted the development of scientific knowledge through the direct experience and involvement of the researchers.

Conceptually, the research approach could be connected to other broader scientific approaches, such as the design science paradigm, widely used in the sphere of information systems. According to a synthesis by Hevner and Chatterjee [21]:

“the design science paradigm has its roots in engineering and the sciences of the artificial. It is fundamentally a problem-solving paradigm. It seeks to create innovations that define the ideas, practices, technical capabilities, and products through which the analysis, design, implementation, and use of information systems can be effectively and efficiently accomplished. Acquiring such knowledge involves two complementary but distinct paradigms, natural (or behavioral) science and design science”.

A researcher using the action research approach does not just observe a process of transformation in a company or organisation but also participates and becomes directly involved in it, acting as a “change agent”. Thanks to their direct involvement, researchers are able to witness the process of change first-hand during the observation–intervention–learning cycle. Knowledge gained during such a process can be enhanced and shared with other companies and researchers [22,23].

At the same time, this approach, based on the direct immersion experience of the researcher, is particularly interesting when studying organisational transformation processes such as those associated with the implementation and deployment of lean management. According to some authors [10,24,25,26,27], there are scarce references to adoption of the action research approach in the search for improvements in health services. This is because the literature mainly centres analysis on case studies (e.g., [4,12,13]). Other papers reflect on, synthesize and review the case studies, looking at them in terms of tools, adopted practices or activities, indicators, affected areas or departments as well as the results obtained (e.g., [9,10,11]). In this context, the approach taken by this paper is not so much to describe why lean management is of interest in the health sector but rather to propose how to implement it successfully, using a scientific methodology that can be replicated and applied in other contexts. At the same time, the literature also identifies the scarcity of applied research into the role played by people in the process improvements [28] which would be linked to the deployment of structured participation systems within the framework of lean management implementation.

**Figure 1:**
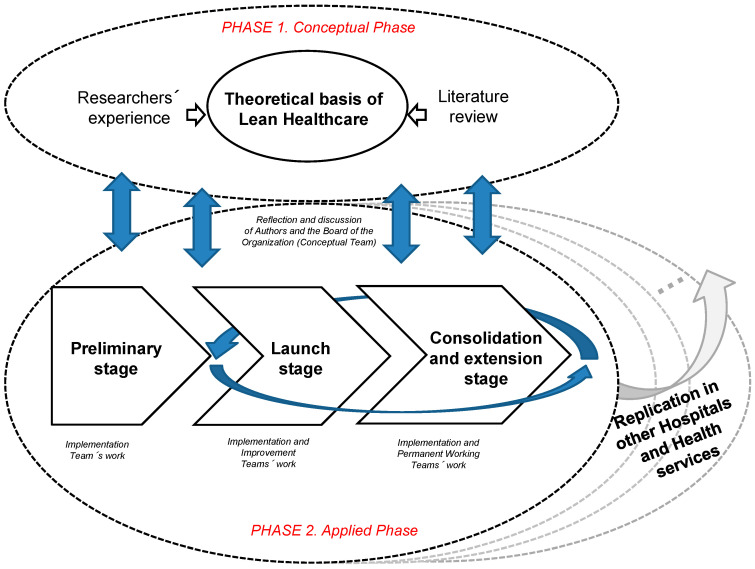
Proposal of the participative methodology.

Näslund et al. [26] propose 3 key points that make research using this approach scientifically relevant: first, deployment of a rigorous, structured, documented system for collaboration between the company (or organisation) and the researchers; second, the significant contribution of the research to the creation of scientific knowledge; and, third, the interest of the company (or organisation) itself in achieving results from the research. Furthermore, companies and organisations also obtain a methodology that can help improve efficiency and performance in their processes in terms of costs, quality, lead times, security, agility, flexibility or sustainability. The scientific interest of this paper is therefore reinforced as no specific references have been found that deal with a participative redesign of processes in the health sector through a lean management program that also adopt an action research approach. In order to apply the action research approach, the authors propose a methodology (see Figure 1) which adapts the two-phase framework (conceptual and applied) proposed by García-Arca et al. [16,29]. That framework, in turn, has its origins in the previous proposals of Coughlan and Coghlan [22], Näslund et al. [26], Coughlan et al. [27] and Farooq and O’Brien [30] which also provide the theory underlying its development.

The first (conceptual) phase includes definition and reflection of the theoretical basis of the proposal for redesigning processes in the health sector by applying lean management principles. This is based on analysis of the literature and the authors’ own experience gained during more than 20 years of implementing lean management and kaizen projects in industrial and service firms. The second (applied) phase involved empirical validation of the theoretical proposal to deploy working teams in different areas, departments or centres. Logically, there is mutual feedback between the two phases. This is a research process with varying levels of involvement and intensity of the researchers depending on the stages within the applied phase (preliminary, launch and consolidation–extension). The authors’ proposal is participative, involving all concerned in the different processes that take place in the area, department or centre under analysis. The participants themselves identify sources of added value for the patient, identify and propose actions to eliminate “waste”, and implement and monitor these actions. Below is an explanation of how each phase was developed.

### 2.1. Phase 1 (Conceptual Phase): Structuring lean Principles in Hhealthcare

**Figure 2:**
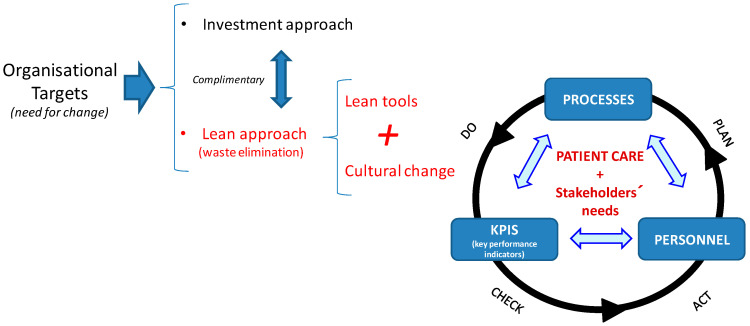
Theoretical basis of the model for implementing lean Healthcare.

Figure 2 shows a synthesis of how the conceptual model proposed in this phase has been developed. The justification for this model is explained in more detail below.

As mentioned in the Introduction (Section 1), firms and organisations are currently subject not only to constant innovation with their products and services but also to demands for increasingly lower prices and increasingly greater standards, deadlines, safety, flexibility and sustainability. This is happening in markets that are increasingly turbulent and volatile and in dynamic environments, particularly at a technological level, which has forced many organisations to seek improved management or redesigned processes, in line with their strategic objectives as a source for their competitive advantage.

This search for design alternatives can be based on investment in technology, equipment or radical innovations but also on small improvements that gradually increase the performance of processes. These two routes should be considered in a complementary fashion and not as mutually exclusive. Without spurning the important impact of the former (the radical route), it can be seen to have some drawbacks, particularly when it comes to having funding available for acquisition or implementation. The latter option, based on small changes that require almost no investment, is the basis for the various approaches, methodologies or philosophies mentioned previously and among which lean management stands out. Traditionally, in the industrial sector the systematic search for alternatives to redesign and improve processes without high investment in technology or equipment form the battleground for all these methodologies [31].

As commented by Hellström et al. [32], when the service sector is compared to the manufacturing or industrial sector, it is widely felt that the service sector, and healthcare in particular, is lagging in terms of adopting new management approaches [33]. For many years it has been considered sufficient for there to be only professional knowledge to ensure quality and safety in the delivery of healthcare services. Today’s healthcare delivery systems are complex; however, calling for further organisational awareness in order to provide the appropriate medical care along the entire patient pathway, generating savings without incurring costs but also improving, for example, the standards of quality, flexibility and safety. Consequently, the problem with healthcare today is largely organisational and not only clinical.

In today’s context, given the complexity in both the nature and the environment of healthcare, managers and staff should analyse, design, and implement improvement processes to achieve efficiency and improve the quality of the provided service. In line with the above, one way of achieving this objective is by means of a management based on lean principles, which will lead to an increase in the performance of hospitals and other health centres.

Lean is a term that was first coined by Womack, Jones and Roos [34] to describe the Toyota Production System (TPS) and the steps to continuously improve the efficiency and effectiveness of a system by driving out waste. They defined lean implementation through five principles that are based on the assumption that organisations are made up of processes. These principles establish that in order to meet its customers’ needs, an organisation must firstly identify what its customers think of as value. Once this is clear, the organisation can work to identify value streams in order to eliminate non-value-adding process steps or waste, make a smooth customer flow in the remaining and value-adding processes, implement pull systems that let the customer pull value from the firm (services should only be provided when the customer downstream asks for them) and to continuously work towards perfection by means of setting ambitious and realistic targets for improvements, as well as to implement mechanisms for process control and continuous improvement.

From an industrial point of view, Ohno defined which aspects should be considered as waste in processes [33]. He identified a total of seven categories of waste: producing too much too early (overproduction), waiting, transportation of people or materials over long distances, duplication or rework, mistakes and errors, unnecessary stock, or non-ergonomic work environments. According to Shah and Ward [35], the concept of lean management can be interpreted from two different points of view. The first of these is the philosophical or cultural perspective relating to fundamental principles and general objectives, such as Womack and Jones’s five principles mentioned above. The second is a more practical perspective which deals with practices or tools that can be more directly applied.

The practices or tools that could be applied in the service sector include, for example, the PDCA cycle (plan, do, check, act), the DMAIC cycle (define, measure, analyse, improve and control), 5Ss, VSM (value stream mapping), standardization, root cause analysis, ABC classification, Ishikawa diagram or visual management activities [36]. The synergetic effect of the application of these practices and tools orientated towards lean principles results in obtaining a high-quality system that offers specific products or services corresponding to client needs, generating little or no waste. Logically, indiscriminate or decontextualized use of these tools or practices without being suitably aligned with the overarching objectives could lead to failure or unnecessary organisational effort.

In summary, one of the chief aims of lean philosophy is to identify and reduce waste throughout the organisation, where waste is defined as any human activity that absorbs resources but creates no value. Simply put, lean means using less to do more. Because lean thinking originated from manufacturing companies, it may be argued that the service sector and especially the healthcare sector may not gain from it. However, Womack and Jones [37] advocate the application of lean thinking in the medical system. They argue that the first step in implementing lean thinking in medical care is to put the patient in the foreground and include time and comfort as key performance measures of the system. Emphasis is given to the promotion of staff participation through multi-skilled teams taking care of the patient and an active involvement of the patient in the process [38].

The term lean healthcare has emerged indicating a stronger focus on efficiency and patient satisfaction within the healthcare sector [39,40], all aligned with the global objectives of the various stakeholders involved. Even if healthcare is specific and cannot be compared directly with other businesses, there is a growing conviction that healthcare can benefit from studying and adapting the theories, principles and methods of lean management, which have proved to be useful in other industries. So, the core values of the Toyota lean method (briefly defined by Liker [41] in The Toyota Way as a long-term philosophy: the right process will produce the right results, add value to the organisation by developing your people and continuously solving root problems which drives organisational learning) are equally applicable to health.

Lean management is a management strategy that is applicable to all organisations, because it has to do with improving processes. All organisations, including healthcare organisations, are composed of a series of processes, or sets of actions intended to create value for those who use or depend on them (customers/patients) [42]. However, the need to focus on the processes is not exclusively addressed by lean management but is also the object of other approaches to management such as BPM (business process modelling) that also seek, in a participative way and with their own visual tools, to model and redesign the activities in a process to make them more efficient [43,44].

Our definition of lean healthcare, based on Dahlgaard et al. [45], is the following: lean healthcare is a management philosophy to develop an internal culture characterised by increased patient and other stakeholder satisfaction through continuous improvements in the processes and activities that create value for them, in which all the interveners in the chain of value of the provided service actively participate in identifying and reducing non-value-adding activities (waste) and promoting the creation of value for the customer/patient across the whole patient flow.

In a perfect process, every step is valuable (creates value for the customer), capable (produces a good result every time), available (produces the desired output, not just the desired quality, every time), adequate (does not cause delay), flexible or agile [12] and linked to continuous flow. Failure in any of these dimensions produces some type of waste. In a healthcare context, this means that all staff are thinking about the principles of value and flow along the patient pathway as an integrated whole and not as a set of independent and isolated functions. This denotes a cultural change or transformation in thinking about the way people do work from a functional perspective to a process perspective [46].

In line with the above, in order to implement lean management in public services, it will first be necessary for there to be some understanding of the principles of lean, in terms of understanding value, focus on flow and pull as well as reduction of waste. Therefore, organisational readiness for implementing lean can be considered in terms of understanding the customer (value), having a process view (value stream), identification of capacity and demand (flow and pull) and linking to strategy, engagement and participation of the staff for problem solving (pursuing perfection), i.e., about understanding what the “value” for the process is, what the process is, what the demand types and patterns are as well as linking the process improvement activity to strategy and finding ways to engage the staff [47]. This initial step will help the organisation to understand the need for change in the way in which they are going about things until that time, so that personnel will feel committed to this approach: by generating a greater added value for the client/patient by reducing/eliminating waste and by implementing continuous improvement.

On the other hand, as stated earlier, process improvement leads to a significant change in culture as it calls for strong leadership, visible support from management and patience (since it is a long-term philosophy). It is vital for senior management to show genuine interest, support and act upon the results delivered and ensure the sustainability of the changes [48,49,50].

Mark Chassin, M.D., president of the Joint Commission, supports hospitals focusing more directly on continuous process improvement to begin to adapt to the principles of so-called high reliability organisations which create tightly defined feedback loops that encourage employees to report minor problems before they rise to the level of errors or lapses in the quality of care provided. He also affirms that:

“*The three critical changes healthcare organizations have to undertake are a leadership commitment to zero major quality failures, the full embodiment and implementation of safety culture and the full deployment of robust process improvement*”.

[51]

When it comes to applying lean, some tools commonly used in healthcare include process mapping, value stream mapping, Kaizen improvement teams, just-in-time process management, and “5S” principles [52,53].

Gowen III and McFadden [54] argue that many healthcare organisations have previously tried to implement lean principles without great success. It normally requires a cultural change where the soft or intangible factors of management (the systemic factors). such as leadership, people management and partnerships, are changed, so that a new organisational culture is developed to support and improve the hospital’s core processes. Empirical research also suggests that the implementation of improvement practices is associated with improved organisational effectiveness, in terms of service quality, customer satisfaction, net cost savings and patient satisfaction [55]. As a means of resolving quality issues, many healthcare organisations have undertaken process improvement (PI) initiatives targeted towards improving organisational performance [56,57].

Nevertheless, Kaplan et al. state that a clear lesson from the current, still early, stage of lean healthcare is that in order to achieve sustainable change results, it is insufficient to simply implement lean tools or practices and that an organisational transformation based on lean principles is required [58]. And Van Rossum et al. argue that appropriate leadership styles and workforce flexibility are success factors in the transition from technical “lean tools” to the required transformation defined as a hospital culture characterised by increased patient and other stakeholder satisfaction through continuous improvement [59]. Finally, Leite et al. analyses the deeper causes that influence the creation of ostensible barriers in healthcare, rather than just focusing on visible elements commonly related to a tools-based approach [60].

The authors believe that, in order to succeed in lean management implementation, these steps must involve three key management pillars for any organisation: (1) processes, (2) KPIS (key performance indicators) and (3) personnel involvement. This approach is consistent with Gowen III and McFadden’s proposal for the successful implementation of improvement programmes [54].

- Processes: An organisation is the sum of interrelated processes aimed at offering a quality, effective and efficient service. The organisation can take direct action on internal processes to improve its results, since these internal processes consume resources and can generate waste or add value for the patient. However, it cannot take direct action on patient needs or the results obtained. In healthcare, many processes require patient involvement.
  Therefore, these processes are key elements to take into account when offering greater customer satisfaction. In order to maximise value and eliminate waste, processes must be evaluated by accurately specifying the value desired by the user, identifying every step in the process and eliminating non-value-added activities, and making value flow from beginning to end based on the pull of the patient [60]; in the jargon of lean management and kaizen, this involves “Go to Gemba”. In this context, the adoption of some Lean tools is useful, particularly, the value stream maps (VSM). Logically, the complexity of the processes being analysed will require more or less diversity in the Lean tools or practices;
- Key performance indicators (KPIS): “What doesn’t get measured doesn’t get managed”. All processes involving change must come with clearly defined goals and objectives, for which there must be well defined indicators. These indicators act as a sort of “mediator” between the system goals and the required actions to achieve these, and thereby become more competitive. These indicators are necessary to measure results and identify deviations from the optimum, as well as trends in values.
  Ultimately, they supply data concerning the system variability and support decisions regarding the taking of preventive or corrective actions. Such indicators will be helpful in determining the current status of the system in terms of effectiveness, efficiency, variability, capacity and quality provided as well as in establishing preventive or corrective actions in the event of detecting deviations from the defined objectives. According to Kissoon [61], it is insufficient to innovate and introduce new processes in healthcare; it is necessary to constantly evaluate the results of the interventions and make the appropriate changes as necessary. Logically, the specific indicators and objectives for the lean management transformation project should be coherent and aligned with the organisation’s overall objectives;
- Personnel involvement: The activities involved in each process are best understood by the personnel, since these processes form part of their daily work routine. Therefore, if they are equipped with the correct tools, they will be able to identify areas for improvement, implement actions and take responsibility for its follow-up and control. For this reason, it is vital to involve personnel from the beginning thereby serving as a motivating factor in their commitment to the process of change. In order to do so, the researchers defined the work organisation for the project based on teams (see next section).

In the conceptual model, the researchers wish to point out that the all actions relating to processes, personnel, and indicators, are all geared towards the implementation of lean management in healthcare, where the primary goal is to improve patient care (and stakeholders’ needs). Likewise, this scheme includes a dynamic vision of the actions that could be implemented which follows the PDCA (plan, do, check, act) continuous improvement cycle. However, in order to develop these conceptual pillars in an applied and participative way, a suitable working system must be designed and adopted during the different stages of implementation. That is the object of the second implementation stage, dealt with below.

### 2.2. Phase 2 (Applied phase): Implementing the Methodology

This second (applied) stage has two basic initial premises that are common to any process of change or transformation: active support from the organisation’s management team and alignment of the lean management programme’s specific objectives with the global ones of the organisation. Logically, without these two basic premises, it is not possible to develop the proposed methodology successfully.

In order to apply and enhance the theoretical basis of our proposal (particularly, personnel involvement), two types of mixed researcher–organisation teams were deployed: conceptual teams and working teams. The conceptual team, which existed throughout the whole project, was a chance for the researchers to meet the Board of the area, department or centre under analysis and design, discuss and reflect on the methodology and its implementation.

Those conceptual meetings were complemented by specific working meetings to define and improve organisation processes with waste reduction and value generation in mind. In practice, they serve as a way of ensuring the participation and commitment of the personnel involved or affected by the process, in a lean management context. In this regard, although structured participation systems for deploying a lean management or continuous improvement program have traditionally been categorized as group systems (e.g., quality circles or improvement groups) or individual systems (e.g., suggestions systems), many authors (the authors included) tend to support group systems because they consider them to be an aid in the development of skills, such as learning, responsibility and communication, between an organisation’s hierarchical levels.

In this context, García-Arca and Prado-Prado [62] propose an organisational structure based on two types of working teams: implementation teams and improvement teams. The job of the implementation team is to define, direct and monitor the continuous improvement process. Given the importance of management involvement, its participation in this team is recommended. The implementation team also decides on the number of working teams, their aims and the times when each one will be launched or wound up. Likewise, this team will select the members of the working teams and track and prioritize the activities they develop. The implementation team stays active throughout the transformation project although its management members may change depending on the area, department or centre in which the improvement teams are being launched.

The improvement teams, meanwhile, are not only responsible for proposing and analysing any problems but also for implementing improvements that contribute towards their objectives (waste reduction and value generation). This they do with supervision from the implementation team, whose management members will be able to facilitate the practical application of proposals made by working team members. The internal transformation process to implement lean management culture in the organisation in this analysis was structured in three differentiated stages: preliminary, launching and consolidation–extension. During the first stage (preliminary), the researchers gained understanding of the processes at the pilot area in the organisation which, in turn, became aware of (and enhanced) the proposed methodology, thanks, in particular, to the conceptual team but also to the implementation team. After the preliminary stage, the launching stage was when the participative methodology was initially implemented in an area, department or centre through improvement teams (typically by using a pilot improvement team).

The consolidation–extension stage had a two-fold objective. First, it reinforced the maintenance (or improvement) of the results obtained in the area, department or centre in which the improvement teams were launched and, second, it encouraged the start-up of new improvement teams in other areas, departments or centres in the organisation so that deployment of lean management and organisational transformation could continue. Everything was overseen by the implementation team. In this context, the improvement teams may be non-permanent (typically in the launch stage in an area, department or centre) or permanent (typically in the consolidation stage of the project in an area, department or centre).

Logically, the meetings held by the different teams must be equipped to function properly if they are to be effective. A review of the recent literature on group participation systems as part of a lean management or continuous improvement program pointed out the importance of providing these teams with a structured working system in order to promote and maintain improvement [2,15,62,63,64,65,66,67,68]. Such a system would include the definition of six points:

- The availability of KPIS for measuring improvement, one of the pillars of our model;
- A preestablished calendar for meetings with dates, start times and lengths. This calendar is usually proposed and justified by the implementation team (not only for their own meetings but for those of the improvement teams), depending on availability and the priority and pace they assign to the project;
- The training program. This program includes both traditional training techniques associated with problem solving, lean tools and an awareness of improvement and teamwork. Some authors recommend complementing this basic training with “learning-by-doing”;
- Communication. This aspect implies the way that actions agreed upon at meetings are documented and communicated, including tasks, responsibilities and deadlines. It can take many forms such as information boards, magazines, intranet, public presentations, etc. For example, the conclusions reached at all the meetings were typically recorded in the minutes, which were sent electronically to the members of the various teams and used for discussion and reflection at the next meeting. Likewise, the main progress, improvements and adopted changes were communicated to the affected areas, departments or centres.
- Resources. These resources were necessary for the proposed improvements to become a reality. A lack of resources available for developing improvements can discourage team members and reduce their commitment to participation programs. In the service sector, particularly in healthcare, the adaptation and suitability of the information system for decision making takes on particular importance.
- Recognition/Reward. This aspect has an important impact on personnel motivation, and consequently on their commitment to lean management projects. Literature differentiates between “reward” (essentially economic), or a “payment in kind”, and “recognition” (essentially social).

The use of the different teams involves more of the people related to processes and is a critical aspect when it comes to enhancing the reflection activity [69,70]. By participating at most of the meetings, the authors became directly involved in the improvement process as agents of change and not just mere observers, which is one of the main strengths of action research. Likewise, the various viewpoints, perspectives and reflections of each of this paper’s authors were shared internally to enrich the methodology. The system adopted for implementing and refining the methodology proposed by the researchers and the organisation does not only lay the foundations for the scientific rigor of this research but also for its future replication in other hospitals, clinics or any other health services.

## 3. Testing the Methodology

The public hospital in which the methodology was applied is one of the largest in Spain. With nearly 4000 workers, it provides healthcare to an area of more than 600,000 inhabitants. A pilot case study was carried out in the sleep unit of this Spanish hospital during a period of eight months. The project was part of the Public Healthcare Innovation Plan and included a total of 14 sub-projects carried out in different areas by different personnel. The Innovation Plan aimed to improve chronic patient care by making the care processes more efficient, agile and secure while minimising human error wherever possible, in line with lean principles. Thus, the lean transformation project was in line with the hospital’s strategic objectives.

### 3.1. Preliminary Stage

During the first stage (preliminary), the researchers gained an understanding of the processes at the hospital which, in turn, they became aware of (and enhanced) the proposed methodology. Therefore, in line with the proposed methodology, a conceptual team was set-up comprising the authors and the Board of the hospital which was particularly motivated by the implementation of lean culture in the organisation. The meetings were held monthly throughout the project. They were kept informed of the project’s progress and emerging changes in which they could possibly be involved, while they also made their own suggestions during the meeting.

At the same time, an implementation team was set-up to guide the work throughout the three stages, particularly oriented towards the pilot area chosen for the launching stage (sleep unit). The implementation team comprised the main managers of the Unit (i.e., the head of the pneumology department and the sleep unit manager) and the researchers. The preliminary stage meant the researchers and practitioners could create an atmosphere of collaboration and trust so that the project could be developed from an action research approach by both the conceptual team and the implementation team.

In this preliminary stage, the implementation team held weekly meetings for one month in order to structure, fine-tune and enhance the proposal from the hospital’s viewpoint by using a general analysis of the sleep unit. Before proposing actions for implementing lean principles (launching stage), the members of the implementation team focused on analysing the sleep unit from the perspective of the model’s pillars (processes, KPIS and personnel involvement): What is the sleep unit? What personnel are involved in patient services? What processes are carried out? What management KPIS (key performance indicators) are currently in place? What objectives do managers wish to achieve with this project?

The sleep unit is a pneumology department specializing in chronic breathing disorders during sleep. There are several types of breathing disorders during sleep, the most prevalent being Sleep Apnea–Hypopnea Syndrome (SAHS). This syndrome is characterised by repeated episodes of obstruction of the upper air tract and occurs when the sleeping patient involuntarily stops breathing. The direct consequences of these episodes are a reduction in oxygen saturation and transitory awakenings. This leads to excessive daytime somnolence, a reduction in quality of life and neurocognitive repercussions. Similarly, daytime somnolence (a key symptom) reduces work performance and increases the possibility of accidents, causing a potentially serious risk for sufferers and third parties.

The prototype of a SAHS sufferer is a middle-aged obese man who snores and is drowsy during the day. Continuous positive airway pressure (CPAP) is the most effective treatment to moderate sleep apnea. Continuous positive airway pressure involves the patient wearing a soft mask over the nose, which is attached to a machine that raises and regulates the pressure of the air that the patient is breathing, preventing the airway from collapsing during sleep. According to figures provided by unit managers, only 20–25% of serious patients in need of treatment have been diagnosed and are receiving treatment. This is due to an increase in the prevalence of this disease among the population in recent years and the dearth of resources available for its analysis, diagnosis and treatment.

This is what led to the project’s main objective: to increase the number of serious patients receiving treatment, accelerating detection, diagnosis and CPAP provision for home treatment. For a patient suffering from SAHS, even more so in a serious case, real added value comes from the treatment itself, since it improves the patient’s standard of living and reduces the mortality rate. Therefore, while the previous steps are necessary for an appropriate treatment, it is important to streamline the process from the first suspicion of the illness to the start of treatment, eliminating any potential obstacles for the patient.

As regards first model’s pillar, the sleep unit used general KPIS which were common to all hospital departments, but not adapted to the specific needs of the unit. By way of example, some of these indicators were: the number of patients discharged per year and the number of consultations carried out per year. Unit managers revealed that they did not carry out any follow-up or control of these indicators, only conveying the end of year figures to verify whether they had met the established yearly objectives. The information system was initially adapted to provide the information for these indicators.

**Figure 3:**
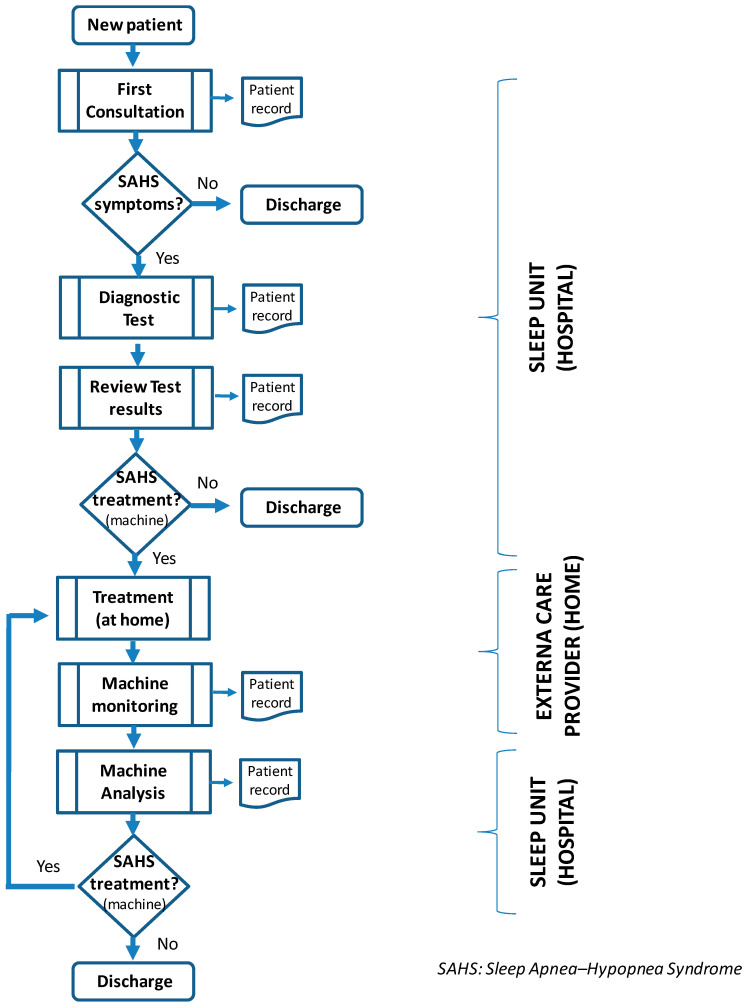
Simplified diagram showing the key processes in patient flow of the sleep unit.

At the same time, within the context of the second of the model’s pillars (processes), generally, a potential sleep unit patient will go through the following steps (see Figure 3):

- A patient with a suspected case of SAHS arrives at the sleep unit following a referral from either a general practitioner (GP) or a specialist;
- At the first consultation, notes are taken of the patient’s parameters and symptoms, and the patient completes a test which is designed to establish the extent of suspected SAHS;
- Following this first consultation, the patient undergoes a diagnostic test in order to determine the frequency of apneas and the seriousness of the illness;
- Once completed, doctors draft a report with the patient’s diagnosis and then they contact the patient to review the test results;
- At this point, the patient may be discharged if the test shows that there is no illness or, alternatively, may start home treatment;
- Treatment requires the patient to sleep while connected to the CPAP, which is supplied by a home care services company specializing in oxygen therapy (hereafter, “external care provider”);
- Periodically, the external care provider supplies machine data to the sleep unit, allowing doctors to make the appropriate adjustments to the machine;
- In the following months, a control test is run on the patient to check his/her development with the treatment. If development is positive, the patient is discharged, otherwise regular checks will be carried out at the hospital. Once the treatment has started, the external care provider company also makes follow-up visits to the patient’s home on a quarterly basis.

There are many parties involved in patient flow (the third pillar of the model), from early clinical suspicion of the existence of the illness to the commencement of treatment and the patient’s subsequent discharge. Each has a different role:

- Those who refer patients to the sleep unit: GPs or specialists;
- Sleep unit personnel: head of the pneumology department, sleep unit manager, doctors, nurses and technicians;
- External care provider: nurses who provide home care during treatment.

### 3.2. Launching Stage

To change this initial situation and deploy the proposed methodology, the implementation team decided to launch a pilot improvement team in the sleep unit. This was the “laboratory” where tests could be carried out to discover the potential and the pitfalls of implementing the methodology on a larger scale in other hospital areas. The improvement team included the different parties involved in the overall patient flow: GPs, doctors and nurses from the unit, nurses from the external care provider company, and the patients themselves. This team also included the researchers and the sleep unit manager, the nexus between the improvement team and the implementation team. The intention was that each working group would contain at least one representative from the parties involved at each stage of the patient flow, including the patient.

This would serve to obtain a global, integrated, internal vision of the value chain of services on offer. In broad terms, the functions of each of the teams were as follows. The improvement team acted directly on the process by gathering data, identifying and analysing problems or opportunities for improvement, putting forward solutions or improvements, implementing these solutions and following-up in order to maintain them over time.

In this stage, the implementation team played a more supporting role by defining the main goals and objectives to achieve, following-up the improvement team actions, and facilitating resources so the improvement team could take action. It is also important to designate a common leader for both working groups who assumes responsibility for the development of the project in the unit. In this stage, the sleep unit manager was designated as the leader, due to the fact of her greater influence and impact on the management of the unit at all levels: planning, personnel management, resource and activity programming and follow-up and control of results. The sleep unit manager also served as a common link between the sleep unit and the hospital managers.

On the basis of the systematics mentioned earlier, the working groups functioned in the following way. Both groups met separately on a weekly basis during the first four months, thereafter they met fortnightly. The implementation team always met before the improvement team, since the implementation team would set the objectives to be met and establish what actions the improvement team needed to carry out. They would also fix a timeframe and assign responsibility for these actions. The improvement team would be informed of any decisions taken by the implementation team. In the improvement team meetings, each member would explain the progress made on the corresponding actions under his/her responsibility. At the end of each meeting, the team members would establish the actions to be carried out before their next meeting.

Between meetings, both working groups dedicated their time to carrying out the assigned tasks. The implementation team meetings lasted around three hours, while the improvement team meetings lasted a maximum of one hour so as to foster a dynamic work environment. All agreements reached in each of the meetings were reflected in a standardised document (minutes) called the improvement action plan (IAP). This contained the following information: date of the meeting, the members present, time and date of the next meeting, agreements reached, a timeframe for action implementation and those responsible for carrying it out.

The researchers began the project with a five-hour training session for members of both working groups. This session served to impart to members a basic knowledge of lean management, process management, problem-solving techniques, and continuous improvement. However, one of its main aims was to make both working groups aware of the importance of their role in the development of the project and, above all, in the improvement of the sleep unit management. As Johnson et al. [71] argue, the training of multidisciplinary project teams can often affect a level of change that no single group or department could implement on its own by breaking down departmental and external silos and opening the lines of communication. The researchers placed special emphasis on the importance of members’ involvement in the detection, implementation, follow-up and control of potential improvements in their daily tasks; through observation, analysis and quantification. The methodology chosen to develop the project was also explained in this training session: the role of each working group, the researcher’s participation in the project, the work dynamic, etc.

In the first meeting of the improvement team, a brainstorming session took place. Thus, attendees were given a blank sheet of paper where, throughout the week, they could note problems or potential improvements that could be made within the sleep unit or in their dealings with the unit. This brainstorming session was complemented with the ideas supplied by the entire hospital’s pneumology department and the members of the implementation team. After a week, 158 ideas for improvement were collected. The implementation team preliminarily evaluated these ideas, and those that were considered viable were analysed in further detail by the improvement team.

Both working groups collaborated in a detailed analysis of the different processes that constitute patient flow, with the implementation team acting as facilitator and the improvement team in an operative role. This analysis sought to identify activities that were wasteful or not value adding, which obstructed the continuous flow of patients through the different processes. It did not look at each stage in isolation, but rather an overall analysis of the value chain. This analysis included observing the processes and the people carrying them out, as well as measuring, where possible, any seemingly wasteful aspects. These wasteful aspects were illustrated in a detailed value stream map (VSM), developing the initial chart described in Figure 3. The VSM was displayed on the wall of the meeting room by the researchers to illustrate the processes, the people implicated at each stage, and any other resources required to provide the service such as documents or databases. This VSM allowed the teams to discuss the current state of the system, the project itself and the viability of carrying out certain actions to improve its management.

Thus, team members were able to identify different activities that did not add value for the patient or which caused rework for themselves, long waits, unnecessary movements, or repeated errors. The waste detected can be classified into two categories: that with a direct impact on the patient (waste which affects the patient directly and conditions his/her satisfaction with the service provided; see Table 1), and that with an indirect impact on the patient (waste that is not felt directly by the patient but impacts negatively on generating added value, and on the effective and efficient management in the Unit; see Table 2).

Once the different sources of waste were identified and pooled together, the implementation team assigned those responsible for carrying out the proposed improvement actions. The aim of these improvement actions was to eliminate waste, to create follow-up and control by means of defined indicators and to continuously improve processes over time by generating new added value. The following improvement actions were carried out to eliminate specific sources of waste grouped into seven categories (see Table 3).

Finally, the members’ effort, commitment, and hard work was recognised in a joint presentation of the results from the experience to the hospital Board, interested parties from other hospital departments, and representatives from the Public Healthcare Innovation Plan. Each working group explained the role they had played in the project and their personal experience. Sleep unit staff expressed their commitment to the project, their responsibility for the KPIS (key performance indicators) values and their current overall vision of the patient flow. They were also satisfied with passing on the knowledge which they had acquired, and with proposing new aspects for improvement. The closing meeting served to showcase the results obtained through the effort, hard work and selfless involvement of both working groups.

### 3.3. Consolidation and Extension Stage

The follow-up and control of the implemented actions is primarily based on the revision and evaluation of the defined indicators, so as to measure how each one is performing. The values obtained from these indicators help to illustrate the effectiveness of the implemented action, since pre-implementation results can be compared with post-implementation results. The frequency with which data is gathered and processed should allow an updated view of the indicators for further follow-up and control. This in turn would allow early detection of any possible deviations in the processes, and any necessary correctional or preventive action to be taken.

The computer application developed greatly facilitates the follow-up, evaluation and control of indicators. The application can pull up reports on indicators, using a database created from all project data that had been inputted since the application was launched. In this way, indicators can be extracted periodically, at the touch of a button. Understanding variability helps healthcare providers to more accurately model and address opportunities for improvement [72] and is the first step to improve a system [73]. Furthermore, McLaughlin [74] argues that variability should be seen as something to be managed and analysed rather than something to be eliminated entirely. To maintain and further enhance the results, the researchers proposed that the sleep unit manager and the head of the pneumology department should meet periodically with sleep unit staff to follow-up the implemented actions and to share ideas on potential improvement areas. In practice, this follow-up process is equivalent to a permanent improvement team. In addition, internal audits were developed to verify the state of the improvements and the key parameter values for the patient flow management.

In any case, it is not enough for one department to streamline processes and improve service if the rest of the organisation is going to do business as usual [75] without progressing in the lean transformation process. Hence, efforts should be made to extend the experience to the rest of the hospital. In this respect, it is especially important to communicate the sleep unit’s achievements to the other hospital departments. Thus, in order to complete the rollout of the methodology to the other units at the hospital, there were training sessions for the rest of the coordinators and managers. The trainers of these sessions were chosen from members of the first two teams.

After these sessions, the coordinator for each unit worked individually to explain the methodology and lean principles to the staff, launching specific improvement teams with similar tasks to the pilot experience. Finally, when the proposed methodology had been deployed, permanent working teams will be adopted to analyse and improve the KPIS in each unit at the hospital, systematically proposing actions for improvement. The system as a whole contributes to the organisational transformation associated with the implementation of lean management within the strategic objectives set by the hospital.

## 4. Results

The applied methodology for lean management implementation was based on the three supporting pillars of processes, personnel involvement and indicators but also on the adoption of an appropriate working system. The resulting improvement actions that were taken led to improvements in the management of patient flow in the sleep unit in terms of quality improvement, costs reduction and productivity. In this regard, the categorisation and prioritisation of patients from the first consultation to the beginning of treatment led to a reduction in waiting time for urgent patients (quality improvement). Waiting time from the first consultation to the diagnostic test showed a reduction of 71.6% with respect to the initial measured values; from the diagnostic test to the start of treatment, the reduction was of 81.6% (see Table 4). Developing the computer application to manage waiting lists systemised the ordering of each different list according to the assigned timeframe for seeing each patient.

The creation of technical instructions, procedures, and protocols allowed process standardisation. This reduced the possibility of human error, made processes more predictable, and therefore easier to manage. The standardisation of processes helped to manage their complexity, allowed the acquisition of new skills, and fostered an understanding of the interrelationship between the different processes that constitute patient flow [76]. The follow-up and control of patients who do not adhere to treatment would result in an increase in treatment efficiency, if the action protocol is approved by the hospital managers. In fact, recalling the CPAP (continuous positive airway pressure) from patients who remain non-adherent after being re-educated in its use would result in a considerable saving of €1.35 per patient per day (costs reduction). Although not fully implemented, the proposed weekly schedule for doctors’ activities served to concentrate activities in continuous periods of time, thereby avoiding the previous case of activities being conducted intermittently throughout the day.

System computerisation allowed the required data for unit management to be captured and stored which, along with patient traceability, reduced the amount of time unit doctors spent manually filling out forms and drafting reports. This, in turn, eliminated duplication in data collection along the patient flow. As a result of database storage, data analysis and indicators, calculation became much simpler and required much less time.

Furthermore, defining specific indicators helped to manage the unit according to its needs and resources, and allowed the systematic identification and resolution of process irregularities, thereby eliminating possible waste. Thus, establishing indicators and corresponding target values brought about significant improvements in productivity. At the end of the project, the number of diagnostic tests carried out had increased by 42.8%, and the number of diagnostic reports drafted had increased by 7.7% (see Table 4). Registering and quantifying the number of unsuccessful diagnostic tests helped identify repetitive human errors and repetitive faults in some machines, which were due to a lack of maintenance.

By the end of the project, 41.5% of the 158 improvement ideas from the brainstorming session had been implemented, 16% were under review, 11.7% were awaiting analysis and the remaining 22.3% were discounted for the time being, since their implementation required further investment.

There were other significant results of a more qualitative nature in relation to achieving competitive advantages. The applied methodology encouraged the involvement of personnel in improving patient services. Personnel were motivated and committed to the initiative. This was reflected in the satisfaction survey that the improvement team completed at the end of the project. One of the most highly valued aspects in this survey was the possibility to propose improvement actions and then implement them. Similarly, many of the improvement team members displayed an interest in participating in more initiatives of this kind.

While developing the methodology for lean management implementation in the unit, some aspects caused greater difficulty than others. It is important to note the following: the complexity of patient flow; the hospital’s internal organisational restructuring that was undertaken in parallel; the unit staff’s day-to-day work that would often prevent them from dedicating time to the project; the lesser involvement and consequent lesser commitment of personnel from outside the unit such as GPs and the external care provider company; and the poorly developed processes related to managing information in the case of data collection, storage, analysis, follow-up and control.

## 5. Discussion

The methodology used in this case study for the preparation and subsequent implementation of lean management gave rise to significant improvements in the efficiency and effectiveness of the entire patient flow. This methodology allowed instant root cause analysis and allowed those participating to feel involved in the change [77]. Sleep Unit staff must take credit for their involvement in the definitive introduction of changes and their maintenance and renewal thus far, as should the pneumology department be thanked for their collaboration. In particular, the sleep unit manager’s participation, commitment, and motivation in leading the implementation of lean management must be gratefully acknowledged. Moreover, the first steps in the introduction of change would not have been possible without the involvement of an external change agent.

In our methodology, this role was undertaken by the researchers, who explained the need for change, provided unit staff with the necessary tools and training to make change, and instilled in them the importance of their involvement in improving the sleep unit, breaking organisational inertia and restraints on change. Logically, the success of the experience is also based on the commitment and support of the hospital management, who aligned the project’s objectives with the global ones; thus, the preliminary stage helped generate a climate of trust and collaboration between the hospital management and the researchers. That climate that was maintained and reinforced during the other stages by the conceptual team.

In the literature there are few detailed examples illustrating the need for collaboration between the worlds of academia and business in a bid to create and validate knowledge within the sphere of health services and lean management. That is why the action research approach adopted here is relevant from a scientific point of view. Such collaboration and knowledge sharing between the hospital and the authors make it possible to qualify and enhance the individual views of each party involved in the research, which underpins the necessary scientific rigor of the action research approach. This approach represents a valuable contribution to scientific literature which goes beyond more technical or theoretic approaches in lean management in healthcare.

As was seen in the case study, for lean management implementation it was not only a matter of analysing each process as an isolated entity, since the greatest complexity and the greatest waste was often found in the links among these processes. Consequently, the greatest potential for improvements is focused on links and functions. By centring the methodology on the processes (“Go to Gemba”, redesigning and/or reviewing tasks), improvement and standardisation are facilitated. Standardisation is key when objectively laying the foundations for capacities, productivities and terms, setting targets and identifying deviations. Process observation and documentation were proven effective, because they can serve as a real eye-opener for staff members who have never before examined their own processes in this way. Since they are immersed in these processes, staff are not normally able to see the surrounding complexity [78]. For example, an important aspect in the case study, both for improving quality and for improving the effectiveness and efficiency of the service, was the definition of three patient categories in the sleep unit (classification). Not all patients have the same diagnosis or are subject to the same risks; consequently, the medical care they receive throughout the patient flow should be appropriate to the seriousness of their symptoms and their personal circumstances.

The selection of the place and time to launch the pilot improvement team (second stage) is also an important element when it comes to planning activities, because it is critical to start with areas, departments or centres that not only ensure success is possible, employing the least resources and lean tools and practices that are not very sophisticated, but also ones that involve workers who are particularly motivated and proactive (in the case study, this was the sleep unit). The good results obtained in these initial experiences are an excellent calling card and incentive to other areas, departments or centres (in the consolidation–extension stage) when launching new improvement teams in more complex settings that require more sophisticated lean tools or practices. The value of these experiences as promotors of transformation or change can be summed up in the common saying: “actions speak louder than words”.

Moreover, the “invisible” hierarchical and functional barriers must be broken down. These are traditionally strong in the healthcare sector, and particularly so in hospitals where there are clearly defined groups (doctors, nurses, administrators, etc.). In order to transform organisations with lean culture, spaces or forums must be created for integration, exchange and collaboration, which is justification for proposing that work be done in a participative way through teams.

The new lean culture means that some roles have changed. For example, managers have become teachers, mentors and facilitators rather than simply directors or controllers. This is especially true for the sleep unit manager who was a key factor in the successful implementation of lean management. Her critical role in the project was brought into sharp focus with the attempted implementation of a proposed weekly schedule for doctors’ activities. Her refusal to take responsibility for this action meant a problematic implementation, taking longer than expected, and eventually not yielding full implementation.

Another important result is the sustainability of lean management, now that the knowledge has been imparted to unit managers and staff. For example, the sleep unit manager now speaks at conferences explaining this case study’s origin, methodology, development, implementation, results and its sustainability. Thus, she is acting as a change agent by sharing this knowledge with sleep unit personnel from other hospitals. In other studies, it has been shown that, following the departure of the consultant, most companies experienced a decrease in improvement. It is crucial, therefore, that the consultant’s lean management knowledge and skills are transferred to the organisation, so that once the consultant leaves, the company has the capability to sustain its lean transformation [79].

This all involves simplifying, rationalising, eliminating bureaucracy and so on. In essence, eliminating waste. In order to do this, an internal climate of trust must be created to encourage each person from within each area, department or centre to leave their comfort zone. In many cases, these personal barriers and settled arrangements are stronger in public organisations than in private ones. In short, internal transformation will have been achieved if lean principles can be inserted into the day-to-day activity of the organisation, not just in specific projects with a start and end date. This is also justification for fostering deployment of permanent teams during the consolidation–extension stage. Likewise, when it comes to programming the activities or priorities of the improvement teams, it is interesting to pay attention not only to activities which impact heavily on patient care and the global objectives of the organisation, but also to activities that can facilitate the work of the people involved in the processes. That helps to increase individual satisfaction and motivation levels and actively contributes to the internal transformation towards lean management attitudes and culture.

As Gowen III & McFadden [54] argued in their study, the intangible factors of management, such as leadership, people management and partnerships, are crucial in the implementation of lean management. Many healthcare organisations failed in implementing lean principles because they were unable to handle these intangibles. In this case study, the intangible factors of unit management were taken into account from the beginning, and the success achieved in implementing actions can be attributed to this. Thus, it is worth noting the satisfaction shown by the members of the various working teams towards the methodology. Such satisfaction promotes staff motivation and commitment to the hospital’s strategic goals, actively helping to improving its efficiency and level of service. Thus, there are three complementary areas in which this satisfaction is clearest [29]: (1) the opening up of a structured, systematic communication channel for analysing and dealing with problems or improvements linked to the processes people were participating in; (2) the possibility of participating directly in the improvement of their working conditions; (3) the possibility of receiving feedback on the quality of work undertaken.

At the same time, the use of KPIS comprised one of the driving forces behind the development, implementation and, in particular, sustainability of lean management. The KPIS keep lean management alive in a way that, in the event of neglect, the management system would fail, and in order to re-initiate it, it would be necessary to start again from the beginning. Furthermore, decision making on management processes should be based on quantified facts that show quantified improvements. If this is not the case, the decisions made may lead to erroneous management and the consequent generation of waste.

In this context of KPIS deployment, adjustment of the information system is of relevance in order to provide or calculate the value of the KPIS. In many cases, such as the one described here, the first teams work with a preliminary information system that acts as a starter, but sooner or later a certain level of “professionalisation” is needed to improve reliability and to connect KPIS to the organisation’s global indicators (with approaches such as the balanced scoreboard). This situation means that some additional resources (not just IT resources) must be foreseen and planned for in order to maintain a widespread lean culture. Given that available resources are always limited, in the worst cases some of the proposed improvements may not be seen as a priority within the global context and could therefore be delayed or even ruled out. When that happens, it must be communicated and explained so as not to cause indifference and demotivation among personnel.

### 5.1. Future Avenues

The analysis of the literature and the methodology proposed in this paper identify lines for applied research for academics and practitioners within the scope of lean management implementation in the health sector. Thus, a lean transformation in any organisation should be addressed with a mid- to long-term perspective. However, the applied experiences in the literature tend to focus on the early stages of this transformation, almost always with an analysis lasting fewer than 24 months (our case study, for example, ran for 8 months).

At the same time, a large number of the papers or reviews found in the literature focus on hospitals; however, there are other, more specific centres (for example, health centres or primary healthcare) that, because of their number, care provision profile and size, could require a specific analysis and adaptation of the methodology when implementing lean principles.

Likewise, benchmarking (internally in each organisation and externally between them) is a little-explored field and can be used to compare and classify practices, results and indicators related to lean management. Logically, given that these KPIS can be different in their conception and implementation, prior work would be required to standardise and bring them into line with a broader quantitative baseline. Such a comparative approach could also be used to identify differences depending on country or region, public or private organisation, or the type of area or department.

### 5.2. Limitations

One significant limitation was the study’s isolated implementation in a single hospital unit. For this reason, we propose that future research should deal with the implementation of this methodology in other hospital departments and health services, so that it can be validated more widely. Furthermore, the area of the hospital chosen to illustrate the launching stage of our methodology, the sleep unit, required unsophisticated lean techniques and tools. In other cases, for application, the diversity and complexity of these tools and indicators would be greater, which would lead to some variety in the effort required in the methodology when it comes to providing the necessary training and resources.

Finally, the ultimate objective of lean management and the proposed methodology is to eliminate waste in the various processes used in health sector organisations; however, some of the improvements (albeit on a small scale) could enter into conflict with the basis for medical or healthcare treatment, the good use of available technologies or even the regulations in force. Logically, in such situations, common sense would have to prevail over any potential for improvement.

## 6. Conclusions

Internal management of healthcare is incredibly complex and a vast quantity of data are collected. In general, it is still not possible to easily identify how a hospital is performing in terms of quality, cost and delivery of services, because a great deal of the information gathered is not linked to measure the efficiency of processes. Thus, it is important to identify what adds value for the patient and what information and activities are necessary in order to provide added value in the best possible way in line with lean principles. The participative methodology offers guidelines to achieve this by focusing on the generation of added value for the patient and eliminating waste in service provision. The proposed methodology could be adapted and adopted by any hospital department, in any other hospital, or in other health services, in other countries. Lean management’s successful implementation would rely on a suitable choice of change agent who can foster change, on good management and coordination of actions related to the three pillars of processes, personnel and KPIS, and on the existence of a true leader in the organisation who would be responsible for the whole process of change.

The advantages of our participative methodology go beyond the important improvement in the hospital unit operations, because people’s commitment and involvement make it possible to bring the organisation into line with lean principles. Likewise, the methodology is participative and proposes support through teams that include people from different functions and hierarchical levels. This across-the-board structure is very important because it allows cohesion, deletes barriers and generates a culture of teamwork and learning. On the basis of what was developed in this paper, it can be said that the main research question was answered affirmatively. That is, it is possible to define and implement a participative methodology that systematically seeks to redesign processes in health services, by applying the scientific action research approach and under a lean management perspective.

## Figures and Tables

**Table 1:** Waste with direct impact on the patient.

Type of Waste	Comments
Long patient waiting lists between processes in the unit	When starting treatment in the sleep unit, the patient must queue for two processes: the diagnostic test and results review. The patient waiting times for each of these processes was measured from the beginning of the project. Corresponding data from the previous year was collected and analysed. In the case of the diagnostic test, the average waiting time for the 211 patients on the waiting list was 67 days, and in the case of the results review, the average waiting time for the 673 patients of the waiting list was 49 days. In both cases there was a high variability in waiting time.
Repetition of diagnostic tests	Some of the monthly diagnostic tests showed an error when analysed on the computer. Those responsible were asked to analyse the results of the diagnostic tests which showed a fault, so as to find its source. At the same time, they registered the diagnostic tests which had been carried out on each of the Unit’s six machines. From this they were able to establish that the most common causes for the fault were: human error due to the patient’s incorrect positioning of the apparatus, a fault in the apparatus itself and a computer fault in downloading the test. During a sample over seven months, there were 482 diagnostic tests, 36 (7.5%) of which were repeated.
Repetitive medical consultations in the unit due to the fact of maladjustment of the CPAP	The mask connecting the patient to the CPAP (continuous positive airway pressure) must be adapted to each patient. Consequently, there were different types of mask. An inadequately fitted mask can cause discomfort while the patient is sleeping, dry mouth, disconnection from the CPAP, or breakage. Before beginning home treatment with the CPAP, the patient is fitted with the most suitable type of mask. However, the patient usually experiences discomfort with the mask after continuous use of the CPAP. Therefore, the majority of return visits to the sleep unit by patients already in treatment are due to maladjustment of the mask. These visits interrupt the scheduled appointments for patients still in the diagnostic stage of the illness.

**Table 2:** Waste with indirect impact on the patient.

Type of Waste	Comments
Delays in drafting diagnostic reports	Before a patient can begin treatment, sleep unit doctors must firstly draft a report on the results of the diagnostic test. In the Unit there were files full of diagnostic test results waiting to be drafted in reports. Managers were unable to estimate how many reports were pending, but the files contained diagnostic test results that were more than one year old. The sleep unit doctors drafted reports whenever they managed to have some free time.
Patients who do not follow the treatment	Unit managers were aware that some patients who had started home treatment were not using the CPAP for the time required for an effective treatment. However, they were unable to estimate how many patients underused the CPAP or the number of patients receiving treatment. The economic cost of each CPAP amounts to €1.35 per day and 15 patients were identified as not having used the machine for more than 10 years. Halfway through the project, 4137 patients in home treatment had been recorded, 15.52% of those used the CPAP for less time than was required for an effective treatment.
Sleep unit specialists dedicating time to activities outside the unit	Sleep unit patients achieve added value through the effective treatment of SAHS. Apart from spending time on activities that add value for patients, doctors and nurses from the sleep unit carry out other activities within the pneumology department such as visiting admitted patients, receiving visits from supervisors, and helping with other activities outside the unit. These activities do not form part of their schedule, and when they arise, doctors and nurses must improvise in order to maintain a minimum service in the sleep unit.
Paper support information system	Initially, all information handled within the unit was on paper, such as the appointments diary, reports on test results, and forms to be filled out by both doctor and patient during the consultation. A large amount of paper-based information was collected from different points along the patient flow which led to the duplication of some data. This complicated data analysis and patient traceability for the unit management.
Difference in criteria for diagnosis and discharge of patients	After pooling the criteria for patient referral to the unit and the criteria followed by unit doctors for patient discharge, there appears to be no uniformity of criteria for decision making.
Impossible to quickly and easily understand different aspects of unit management	Essential aspects of unit management such as knowing the current workload, the traceability of each patient, or the productivity of the unit, were difficult if not impossible for the unit specialists to understand. This was due to a lack of defined indicators and a lack of information to create them.

**Table 3:** Proposed improvement actions.

Type of Improvement	Comments
Categorisation and prioritisation of patients according to seriousness	Three different patient categories were defined depending on the seriousness of the illness, the risk of accident due to the symptoms of the illness and the patient’s profession (the risk of accident is higher if a professional driver suffers from drowsiness, rather than an administrator). The categories of urgent, preferential and normal were established. The appropriate category is assigned to the patient by sleep unit professionals in the first consultation. Any patient diagnosed as urgent is always given priority in the flow. This action is similar to the triage method used in emergency departments. This method efficiently rations patient treatment when there are insufficient resources for all to be treated immediately. The sleep unit has a large number of patients on waiting lists. Since it is not possible to attend to all patients at once, priorities were established for administering medical care.
Drafting technical instructions	Technical instructions were drawn up for all activities where the probability of human error occurring was greater. The instructions explained the optimal way to carry out these activities. In the case of the diagnostic test apparatus, step-by-step instructions were drawn up for the positioning of each part of apparatus. Each step contained a small photo with an explanatory text. These technical instructions were posted on the wall in the sleep unit and placed in the bags that contained the diagnostic test apparatus.
Action procedure for patients who do not adhere to treatment	An action procedure was drawn up in conjunction with the external care provider company to identify patients that voluntarily underused the CPAP (continuous positive airway pressure). Under this procedure, the external care provider company would report a monthly list of non-adherent patients (including their reasons for not using the CPAP) and the sleep unit would evaluate each particular case. By way of example, for a patient who used the CPAP, but not sufficiently, efforts would be made to re-educate the patient in its use. In the case of a patient who did not use the CPAP at all or refused to use it despite having been re-educated in its use, sleep unit doctors would have the necessary authority to recall the CPAP.
Categorisation and prioritisation of doctors’ activities	The daily activities carried out by unit doctors were recorded over a three-week period. These activities were classified into three categories: key activities that impact patients directly, such as consultations; general activities that impact patients indirectly, such as training sessions for unit staff; and other activities that do not add value for the patient, such as meeting unit visitors. Target percentages for the time dedicated daily to each type of activity were established: 70% for key activities, 20% for general activities, and 10% for the rest. In addition, a proposed weekly schedule for doctors’ activities was drawn up.
Computerisation of data capture, storage and analysis	During the case study, an “Access” computer application was developed to capture, store and analyse all data handled within the sleep unit. This includes doctor and patient forms, medical reports, test results and so on. Data capture, analysis, treatment, and storage would be centralised in a single application and database. The patient appointment process was also systemised. Each patient was automatically allocated the maximum admissible time within which he/she should be seen (by appointment), in line with the patient’s previously assigned category of urgent, preferential or normal. The patients on the waiting list were arranged from most to least urgent according to this maximum admissible time for receiving an appointment. Furthermore, if the patient is not seen within this maximum admissible time, a notice is sent to the person in charge of managing the appointment process.
Development of internal procedures and protocols	Internal procedures and protocols were drafted in order to standardise processes that were carried out by different doctors. This sought to homogenise diagnosis and treatment criteria for patients, reduce the probability of producing human error, and facilitate the task of estimating productivity, quality and efficiency indices.
Defining indicators that are linked to objectives	Indicators were defined for the appropriate management of each value adding activity. These indicators were established to measure the following: unit productivity, such as the number of consultations per week and number of reports drafted per week; quality, in terms of waiting time to access the system, waiting time between processes, total accrued patient waiting time, rate of test repetition and number of patients not adhering to treatment; system load, as in the number of patients in the system, number under treatment compared to the number of diagnostic tests and number of patients suffering from or with symptoms of the illness. Depending on the situation, either a target value or an admissible value was established for each indicator.

**Table 4:** Some of the KPIS defined in the case study with their initial and current average values.

Type of KPI (Key Performance Indicators)	Description			Initial Average Value	Current Average Value	Variation (%)
Productivity	Number of first consultations per week			20	20	-
	Number of diagnostic tests per week			14	20	42.8%
	Number of reports drafted per week			26	28	7.7%
Quality	Waiting time for the patient (days)	From first consultation to diagnostic test	Urgent	67	19	71.6%
			Preferential		62	7.5%
			Normal		148	−121%
		From diagnostic test to results review	Urgent	49	9	81.6%
			Preferential		15	69.3%
			Urgent		30	38.7%
